# Supplementary material for: Two locus inheritance of non-syndromic midline craniosynostosis via rare SMAD6 and common BMP2 alleles
Source: eLife. 2016 Sep 8;5:e20125. doi: 10.7554/eLife.20125 (PMC5045293; doi:10.7554/eLife.20125)
Supplement: Figure 2—source data 1. — Highlighted variants indicate de novo mutations; 'D' and 'T' respectively denote damaging and tolerated missense variants called by MetaSVM. DOI: http://dx.doi.org/10.7554/eLife.20125.007 [file elife-20125-fig2-data1.docx]

| **Patient** | **Position on Chromosome 15** | **Ref** | **Alt** | **Impact** | **ExAC03**  **Frequency** | **MetaSVM** |
| --- | --- | --- | --- | --- | --- | --- |
| MET111-1P,2P | 67073775 | C | T | R465C | 9.38 x 10^-6^ | D |
| MET115-1P | 67073851 | T | C | I490T | Novel | D |
| MET127-1P | 66996263 | C | T | Q223* | Novel | Stop |
| MET148-1P | 67073350 | C | T | P323L | Novel | D |
| MET153-1P | 67073550 | G | T | G390C | Novel | D |
| MET154-1P,2P | 67073601 | G | T | E407* | 9.00 x 10^-6^ | Stop |
| MET179-1P | 66995977 | TC | del | S130fs*146 | Novel | Frameshift |
| SAGMET100-1P | 67073437 | A | AAT | A353fs*187 | Novel | Frameshift |
| SAGMET101-1P | 66995828 | 19bp | del | Q78fs*41 | Novel | Frameshift |
| SAGMET107-1P | 67004047 | G | A | E287K | 3.30 x 10^-5^ | D |
| SAGMET104-1P,2P | 67073416 | G | del | R345fs*194 | Novel | Frameshift |
| SAG158-1P | 67073502 | G | T | E374* | Novel | Stop |
| SAG210-1P | 67008800 | A | G | T306A | Novel | D |
| SAG220-1P | 67004027 | T | TT | R281fs*13 | Novel | Frameshift |
| SAG-PROBAND3 | 66995873 | A | T | M93L | Novel | T |

**Figure 2- Source Data 1.** **Variants identified in *SMAD6*.** Highlighted variants indicate *de novo* mutations; “D” and “T” respectively denote damaging and tolerated missense variants called by MetaSVM.
