# Supplementary material for: Two locus inheritance of non-syndromic midline craniosynostosis via rare SMAD6 and common BMP2 alleles
Source: eLife. 2016 Sep 8;5:e20125. doi: 10.7554/eLife.20125 (PMC5045293; doi:10.7554/eLife.20125)
Supplement: Figure 2—source data 2. — Source Data for Figure 2—figure supplement 2. DOI: http://dx.doi.org/10.7554/eLife.20125.008 [file elife-20125-fig2-data2.docx]

| Gene | DNA Change | Impact | Forward Oligonucleotide | | Reverse Oligonucleotide | Annealing Temp (°C) | |
| --- | --- | --- | --- | --- | --- | --- | --- |
| *SMAD6* | c.232_250del | Q78fs | CGCTGAGGGAACGGACCCCCGG | CCCGCAGCTGCGCCGACCCGCAGTG | | 56 |  |
| *SMAD6* | c.A277T | M93L | CGCTGAGGGAACGGACCCCCGG | CCCGCAGCTGCGCCGACCCGCAGTG | | 56 |  |
| *SMAD6* | c.381_382delTC | S130fs | CGCTGAGGGAACGGACCCCCGG | CCCGCAGCTGCGCCGACCCGCAGTG | | 56 |  |
| *SMAD6* | c.C667T | Q223* | CGCTGAGGGAACGGACCCCCGG | CCCGCAGCTGCGCCGACCCGCAGTG | | 56 |  |
| *SMAD6* | c.839_840insT | R281fs | GGTCACACAGCCAAAGGTGGC | GCTGGGTCTGCTTTGGCCCAAC | | 56 |  |
| *SMAD6* | c.G859A | E287K | GGTCACACAGCCAAAGGTGGC | GCTGGGTCTGCTTTGGCCCAAC | | 56 |  |
| *SMAD6* | c.A916G | T306A | GGTCACACAGCCAAAGGTGGC | GCTGGGTCTGCTTTGGCCCAAC | | 56 |  |
| *SMAD6* | c.C968T | P323L | CAACCTGGCACACGGTGCCCAC | GGTGGCGGTGGCCGCGGCCTCCC | | 56 |  |
| *SMAD6* | c.1034delG | R345fs | CAACCTGGCACACGGTGCCCAC | GGTGGCGGTGGCCGCGGCCTCCC | | 56 |  |
| *SMAD6* | c.1055_1056insAT | A353fs | CAACCTGGCACACGGTGCCCAC | GGTGGCGGTGGCCGCGGCCTCCC | | 56 |  |
| *SMAD6* | c.G1120T | E374* | CAACCTGGCACACGGTGCCCAC | GGTGGCGGTGGCCGCGGCCTCCC | | 56 |  |
| *SMAD6* | c.G1168T | G390C | CAACCTGGCACACGGTGCCCAC | GGTGGCGGTGGCCGCGGCCTCCC | | 56 |  |
| *SMAD6* | c.G1219T | E407* | CAACCTGGCACACGGTGCCCAC | GGTGGCGGTGGCCGCGGCCTCCC | | 56 |  |
| *SMAD6* | c.C1393T | R465C | CAACCTGGCACACGGTGCCCAC | GGTGGCGGTGGCCGCGGCCTCCC | | 56 |  |
| *SMAD6* | T1469C | I490T | CAACCTGGCACACGGTGCCCAC | GGTGGCGGTGGCCGCGGCCTCCC | | 56 |  |
| *SMURF1* | c.C1402T | R468W | CAGGATCCACACCAAGCTCTTATGC | GGGACACTTTTCCCAAGAAGC | | 56 |  |
| *SPRY1* | c.16delC | Q6fs | GGATTTCAGATGCATGCCAGG | CATAGTCTAATCTCTGACGGC | | 56 |  |
| *SPRY4* | c.G478T | E160* | GACCCAGCAGGAAGGCAACG | GGTCCAGCGGCTGGCAGTGGACC | | 56 |  |
| *BMP2* | rs1884302 | C/T | GGTGGAAGGTGAAGGGTCCC | GAGTGAGAATATAAGGTATTGC | | 56 |  |
| *BBS9* | rs10262453 | A/C | CCCAAACCTAGGCCTCAGTCCTATCC | GCCCACTGGTTTGTTACCTTG | | 56 |  |

**Figure 2- Source Data 2**. **PCR primer sequences for Sanger sequencing of reported variants.** Source Data for Figure 2- Figure Supplement 2.
