# Supplementary material for: Two locus inheritance of non-syndromic midline craniosynostosis via rare SMAD6 and common BMP2 alleles
Source: eLife. 2016 Sep 8;5:e20125. doi: 10.7554/eLife.20125 (PMC5045293; doi:10.7554/eLife.20125)
Supplement: Figure 3—source data 1. — DOI: http://dx.doi.org/10.7554/eLife.20125.013 [file elife-20125-fig3-data1.docx]

**Figure 3- Source Data 1.** Source data for Figure 3- Figure Supplement 3.

**Burden of rare, (allele frequency < 2 x 10^-5^) *de novo* and transmitted damaging (LOF + damaging missense) variants comparing European craniosynostosis cases to European controls (autism parents, NHLBI ESP, ExAC03).**

LOF + D-mis variants showing P < 0.005 vs. all control groups.

|  | CASES | | |  | AUTISM CONTROLS | | | |  | NHLBI CONTROLS | | | |  | | EXAC03 CONTROLS | | | | | |
| --- | --- | --- | --- | --- | --- | --- | --- | --- | --- | --- | --- | --- | --- | --- | --- | --- | --- | --- | --- | --- | --- |
| GENE | VAR | | REF |  | VAR | REF | P | OR |  | VAR | REF | P | OR |  | VAR | | REF | | P | | OR |
| *SMAD6* | | 8 | 330 |  | 6 | 6,668 | 6.26 x 10^-8^ | 26.9 |  | 6 | 8,588 | 9.54 x 10^-9^ | 34.7 |  | 41 | | | 59,329 | | 3.58 x 10^-10^ | 35.1 |
| *PIK3C2A* | | 4 | 340 |  | 8 | 6,666 | 2.05 x 10^-3^ | 9.8 |  | 8 | 8,578 | 8.34 x 10^-4^ | 12.6 |  | 46 | | | 66,584 | | 1.31 x 10^-4^ | 17.0 |

VAR, # of variant alleles; REF, # of reference alleles; P, two-sided P-value by Fisher’s exact test; OR, odds ratio. Reference allele counts from NHLBI and ExAC represent the median number of European and non-Finnish European alleles respectively across all reported variants for each gene. *SMAD6* burden in this case-control analysis excludes exon 1, as this exon was well-covered in cases but poorly captured in controls. This resulted in exclusion of 3 damaging variants identified in cases.

**Burden of rare, (allele frequency < 2 x 10^-5^) *de novo* and transmitted LOF variants comparing European craniosynostosis cases to European controls (autism parents, NHLBI ESP, ExAC03).**

LOF variants showing P < 0.005 vs. all control groups.

|  | CASES | |  | AUTISM CONTROLS | | | |  | NHLBI CONTROLS | | | |  | | EXAC03 CONTROLS | | | | | |
| --- | --- | --- | --- | --- | --- | --- | --- | --- | --- | --- | --- | --- | --- | --- | --- | --- | --- | --- | --- | --- |
| GENE | VAR | REF |  | VAR | REF | P | OR |  | VAR | REF | P | OR |  | VAR | | REF | | P | | OR |
| *SMAD6* | 4 | 334 |  | 0 | 6,674 | 5.68 x 10^-6^ | - |  | 1 | 8,593 | 9.78 x 10^-6^ | 102.6 |  | 5 | | | 59,365 | | 1.24 x 10^-7^ | 141.9 |

VAR, # of variant alleles; REF, # of reference alleles; P, two-sided P-value by Fisher’s exact test; OR, odds ratio. Reference allele counts from NHLBI and ExAC represent the median number of European and non-Finnish European alleles respectively across all reported variants for each gene. *SMAD6* burden in this case-control analysis excludes exon 1, as this exon was well-covered in cases but poorly captured in controls. This resulted in exclusion of 3 damaging variants identified in cases.
