# Supplementary material for: Two locus inheritance of non-syndromic midline craniosynostosis via rare SMAD6 and common BMP2 alleles
Source: eLife. 2016 Sep 8;5:e20125. doi: 10.7554/eLife.20125 (PMC5045293; doi:10.7554/eLife.20125)
Supplement: Supplementary file 1. — (A) Exome Sequencing Quality Statistics for all members of craniosynostosis kindreds (n = 455) and autism controls (n = 3337). (B) TDT of an intergenic BMP2 risk allele and intronic BBS9 risk allele in SMAD6 mutation carriers with craniosynostosis. (C) Optimized two locus and single locus parametric models of genotype specific penetrances for SMAD6 and BMP2. (D) Family specific lod scores for each kindred under the two locus and single locus models. (E) Clinical features and BMP2 genotypes in craniosynostosis patients with rare SMAD6, SMURF1, SPRY1, or SPRY4 mutations. (F) De novo mutations identified per trio. DOI: http://dx.doi.org/10.7554/eLife.20125.023 [file elife-20125-supp1.docx]

**Two locus inheritance of non-syndromic midline craniosynostosis via rare *SMAD6* and common *BMP2* alleles**

Andrew T. Timberlake^1-3^, Jungmin Choi^1,2^, Samir Zaidi^1,2^, Qiongshi Lu^4^, Carol Nelson-Williams^1,2^, Eric D. Brooks^3^, Kaya Bilguvar^1,5^, Irina Tikhonova^5^, Shrikant Mane^1,5^, Jenny F. Yang^3^, Rajendra Sawh-Martinez^3^, Sarah Persing^3^, Elizabeth G. Zellner^3^, Erin Loring^1,2,5^, Carolyn Chuang^3^, Amy Galm^6^, Peter W. Hashim^3^, Derek M. Steinbacher^3^, Michael L. DiLuna^7^, Charles C. Duncan^7^, Kevin A. Pelphrey^8^, Hongyu Zhao^4^, John A. Persing^3^, Richard P. Lifton^1,2,5,9^

^1^Department of Genetics, Yale University School of Medicine, New Haven, CT, USA

^2^Howard Hughes Medical Institute, Yale University School of Medicine, New Haven, CT, USA

^3^Section of Plastic and Reconstructive Surgery, Department of Surgery, Yale University School of Medicine, New Haven, CT, USA

^4^Department of Biostatistics, Yale University School of Medicine, New Haven, CT, USA

^5^Yale Center for Genome Analysis, New Haven, CT, USA

^6^Craniosynostosis and Positional Plagiocephaly Support, New York, NY, USA

^7^Department of Neurosurgery, Yale University School of Medicine, New Haven, CT, USA

^8^Child Study Center, Yale University School of Medicine, New Haven, CT, USA

^9^The Rockefeller University, New York, NY, USA

SUPPLEMENTARY FILE 1

**Supplementary File 1A.**

**Exome Sequencing Quality Statistics for all members of craniosynostosis kindreds (n=455) and autism controls (n=3,337).**

|  | Cases | Controls |
| --- | --- | --- |
| Read length (bp) | 74 | 74 |
| Number of reads per sample (M) | 81.30 | 115.29 |
| Percentage of targeted bases with more than 8 independent reads (%) | 94.68 | 94.88 |
| Percentage of targeted bases with more than 20 independent reads (%) | 83.76 | 86.70 |

**Supplementary File 1B.**

**TDT of an intergenic *BMP2* risk allele in *SMAD6* mutation carriers with craniosynostosis.**

|  | Transmitted | Non-transmitted |
| --- | --- | --- |
| Craniosynostosis (+) | 11 | 2 |
| Expectation | 6.5 | 6.5 |

P=0.013 via Chi square with 1 Df.

Transmission disequilibrium test of *BMP2* risk locus rs1884302 allele ‘C’ in 13 kindreds with rare, damaging *SMAD6* variants.

**TDT of an intronic *BBS9* risk allele in *SMAD6* mutation carriers with craniosynostosis.**

|  | Transmitted | Non-transmitted |
| --- | --- | --- |
| Craniosynostosis (+) | 4 | 10 |
| Expectation | 7 | 7 |

P=0.89 via Chi square with 1 Df.

Transmission disequilibrium test of *BBS9* risk locus rs10262453 allele ‘A’ in 13 kindreds with rare, damaging *SMAD6* variants.

**Supplementary File 1C.**

**Optimized two locus and single locus parametric models of genotype specific penetrances for *SMAD6* and *BMP2***

| **Genotype**  **(*SMAD6*; *BMP2*)** | **Penetrance in**  **two locus model (*SMAD6*/*BMP2*)** | **Penetrance in**  **single locus model**  **(*SMAD6*)** |
| --- | --- | --- |
| +,D; C,C | 1 | 0.2 |
| +,D; C,T | 1 | 0.2 |
| +,D; T,T | 0.09 | 0.2 |
| +,+; C,C | 0.0032 | 0.0032 |
| +,+; C,T | 0.0008 | 0.0008 |
| +,+; T,T | 0.0002 | 0.0002 |

Alleles at *SMAD6* (+ or D) are wild-type or have rare damaging variants, respectively. For alleles of a common SNP near *BMP2*, rs1884302, which is associated with midline craniosynostosis^15^, allele C confers increased risk. Penetrance estimates under the maximum lod score models are shown. Both models specify a phenocopy rate of 0.0002 (based on a prevalence of 1/4000 for nonsyndromic midline craniosynostosis, with 7% of these cases attributable to *SMAD6* + *BMP2* risk alleles as well as a 4- fold increase in risk conferred by each *BMP2* risk allele based on the previous GWAS^15^.

**Supplementary File 1D.**

**Family specific lod scores for each kindred under the two locus and single locus models.**

| **Kindred** | **Lod score, two locus model** | **Lod score, single locus model** |
| --- | --- | --- |
| Q78fs*41 | .564 | .300 |
| R345fs*194 | .220 | .645 |
| A353fs*187 | 1.01 | .548 |
| R281fs*13 | 0.564 | 0.300 |
| T306A | 0.682 | 0.295 |
| E374* | NA | NA |
| S130fs*146 | 0.300 | 0.301 |
| Q223* | 0.662 | 0.249 |
| P323L | 0.564 | 0.300 |
| G390C | NA | NA |
| E407* | 1.13 | 0.600 |
| R465C | 0.743 | 0.344 |
| I490T | 0.938 | 0.340 |
| **Total lod score** | **7.37** | **4.22** |
| **Odds ratio in favor of linkage** | **2.3 x 10^7^:1** | **1.7 x 10^4^:1** |

Lod scores under the parametric models specified in Supplementary File 1C are shown for each kindred harboring a *SMAD6* variant. Kindreds with *de novo* mutation in the proband are not informative for segregation (NA). The two locus model is >1,400x more likely than the single locus model under the optimized models.

**Supplementary File 1E.**

**Clinical features and *BMP2* genotypes in craniosynostosis patients with rare *SMAD6*, *SMURF1*, *SPRY1*, or *SPRY4* mutations.**

| Kindred ID | Age (years) | Sex | Age at surgery | Suture(s) Involved | Mutation | *BMP2* Genotype | Other Phenotypes |
| --- | --- | --- | --- | --- | --- | --- | --- |
| SAGMET107-1 | 12 | M | 2.5yrs- late diagnosis | Sagittal and metopic | *SMAD6*: E287K, transmitted | C/C | -poor reading and spelling abilities, held back two years, frequent headaches, frequent complaints of bony thoracic pain |
| SAGMET101-1 | 1 | M | 4 months | Sagittal and metopic | *SMAD6*: Q78fs*41 transmitted,  *de novo* in mother | T/C | -Meeting all milestones |
| SAGMET104-1  SAGMET104-2 | 5  1 | M  M | 6 months  5 months | Metopic  Sagittal | *SMAD6*: R345fs*194, transmitted  *SMAD6*: R345fs*194, transmitted | T/T  T/C | -No delays  -Meeting all milestones |
| SAG158-1 | 6 mos | M | 2.5 months | Sagittal | *SMAD6*: E374*,  *de novo* | C/C | -Infant, hypospadias |
| SAGMET100-1  SAGMET100-2 | 2  30 | M  F | 4 months  No surgery | Sagittal and metopic  Sagittal and metopic | *SMAD6*: A353fs*187, transmitted  *SMAD6*: A353fs*187, transmitted | T/C  T/C | -Speech delay, early intervention for neurodevelopmental delay  -Lifelong headaches, some delays |
| MET127-1 | 5 | F | 5 months | Metopic | *SMAD6*: Q223*, transmitted | T/C | -Speech delay, gross motor delay, fine motor impairment, language delays, persistent delays at age 5, asthma |
| MET148-1 | 6 mos | M | 3 months | Metopic | *SMAD6*: P323L, transmitted | T/C | -Infant |
| MET115-1 | 5 | M | 6 months | Metopic | *SMAD6*: I490T, transmitted | T/C | -Speech and motor delays, ankyloglossia, lip tie, inguinal hernia, retractile testis, asthma |
| MET111-1  MET111-2 | 15  15 | M  M | 1 year  1 year | Metopic  Metopic | *SMAD6*: R465C, unknown  *SMAD6*: R465C, unknown | T/C  T/C | - Global developmental delay, sensory disorder, marked speech delay, ADHD, inguinal hernia  - Global developmental delay, sensory disorder, marked speech delay, ADHD, inguinal hernia |
| MET154-1  MET154-2 | 6  6 | M  M | 6 months  opted against | Metopic  Metopic | *SMAD6*: E407*, transmitted  *SMAD6*: E407*, transmitted | T/C  T/C | -Early speech and motor delay  -Early speech and motor delay |
| Kindred ID | Age (years) | Sex | Age at surgery | Suture(s) Involved | Mutation |  | Phenotype |
| MET153-1 | 4 mos | F | 2.5 months | Metopic | *SMAD6*: G390C, *de novo* | T/T | -Infant |
| MET179-1 | 6 | M | 8 months | Metopic | *SMAD6*: S130fs*146, transmitted | T/T | -Persistent motor delays |
| SAG220-1 | 7 | M | 17 months (late diagnosis) | Sagittal | *SMAD6*: R281fs*13, transmitted | T/C | -No delays |
| SAG210-1 | 3 | F | 4 months | Sagittal | *SMAD6*: T306A, transmitted | T/C | -Speech delay until age 2.5 |
| SAGFAMILY1-1  SAGFAMILY1-2 | 9  5 | F  M | 4 months  4 months | Sagittal  Sagittal | *SPRY1*: Q6fs*8,  *de novo* in mother  *SPRY1*: Q6fs*8,  *de novo* in mother | C/C  C/C | -No other medical history  -Wolf-Parkinson-White syndrome evident at birth, recurrent episodes of V-Tach requiring cardioversion, ankyloglossia, lip tie |
| SAG150-1 | 9 mos | M | 7 months | Sagittal | *SPRY4*: E160*,  *de novo* | C/C | -Infant |
| MET149-1 | 4 | F | 6 months | Metopic | *SMURF1*: R468W, *de novo* | T/C | -No other medical history |
|  |  |  |  |  |  |  |  |

*De novo* mutations are indicated. The primary inclusion criterion was LOF and damaging missense with allele frequency < 2 x 10^-5^ ; for completeness, the E287K variant with slightly higher allele frequency is included. For *BMP2* genotypes, alleles at rs1884302 are shown. The ‘C’ allele predisposes to midline craniosynostosis compared to the ‘T’ allele^15^.

**Supplementary File 1F.**

**
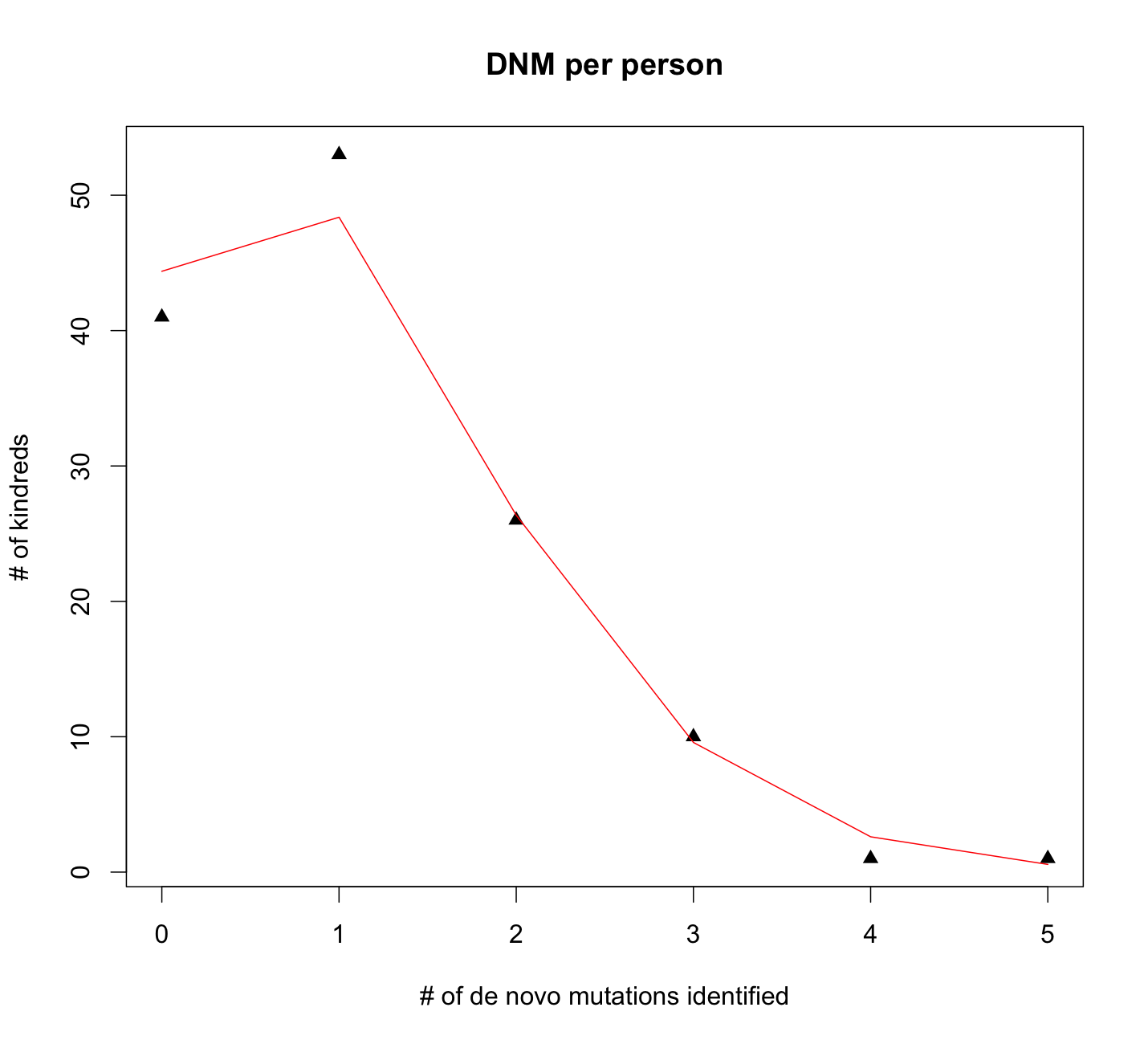
**

***De novo* mutations identified per trio.** The number of *de novo* mutations identified per proband via exome sequencing of 132 case-parent trios was plotted (black triangles) alongside the expected Poisson distribution (red curve), demonstrating that the observed number of *de novo* mutations per proband closely matches expectation.
